# Multi-omics pan-cancer analysis reveals the prognostic values and immunological functions of PPA2, with a spotlight on breast cancer

**DOI:** 10.3389/fimmu.2024.1435502

**Published:** 2024-08-08

**Authors:** Jia-Ning Zhang, Bei-Bei Yang, Lin-Wei Li, Hao Xu, Bin Wang, Zi-Lu Yi, Xi-Rui Zhou, Hong Liu

**Affiliations:** ^1^ The Second Surgical Department of Breast Cancer, Tianjin Medical University Cancer Institute & Hospital, National Clinical Research Center for Cancer, Tianjin, China; ^2^ The Second Surgical Department of Breast Cancer, Tianjin’s Clinical Research Center for Cancer, Tianjin, China; ^3^ Key Laboratory of Breast Cancer Prevention and Therapy, Tianjin Medical University, Ministry of Education, Tianjin, China

**Keywords:** PPA2, pan-cancer analysis, multi-omics, immunological function, breast cancer

## Abstract

**Background:**

Recently, the role of inorganic pyrophosphatase 2 (PPA2) has been remaining merely superficial in many tumors. Hence, the aim was to analyze the potential functions of PPA2 in pan-cancer, focusing on its role in breast cancer.

**Methods:**

A systematic pan-cancer analysis conducted primarily utilizing various open databases such as TCGA and GTEx. We explored the clinical value of PPA2 as well as various biological functions, including expression levels and subcellular localization, multi-dimensional immune-correlation analysis, co-expression networks, and gene heterogeneity. In addition, we not only verified the function of PPA2 through cell experiments but also analyzed PPA2 at the single-cell level and its drug sensitivity.

**Results:**

PPA2 is abnormally expressed in various tumors, and it is mainly distributed in mitochondria. Furthermore, the indicators (OS, DSS, DFI, and PFI) of analysis hint that PPA2 exhibits significant prognostic value. At the same time, the genomic heterogeneity (including TMB, MSI, MATH, and NEO) of PPA2 in pan-cancer was analyzed. Across multiple tumors, the results showed a close correlation between PPA2 expression levels and different immune signatures (such as immune cell infiltration). All of these indicate that PPA2 could potentially be applied in the guidance of immunotherapy. We also have demonstrated that PPA2 promoted the process of breast cancer. Finally, some potential therapeutic agents (such as Fulvestrant) targeting the abnormal expression of PPA2 are revealed.

**Conclusion:**

In conclusion, the results demonstrated the great value of PPA2 in pan-cancer research, as well as its potential as a therapeutic target for breast tumors.

## Introduction

Among all kinds of diseases, malignant tumor is the first killer threatening human health (1). To conquer malignant tumors, human beings try to seek a variety of different treatment modes. With the advent of the era of tumor precision therapy, the same disease but different patients may use different treatment measures (2–4). While researching various tumor treatments, it is important to identify commonalities and discover effective therapeutic drugs or targets shared among different types of tumors. For example, TP53 is a tumor suppressor gene that is mutated in a variety of tumors, such as breast cancer, liver cancer, non-small cell lung cancer (5–8). At this point, although the tumor type is different, we can treat it with the same drug that targets the same mutated gene.

Inorganic pyrophosphatase 2(PPA2) is a pyrophosphatase that hydrolyzes inorganic pyrophosphate (PPi) from many nucleotide-dependent reactions into two orthophosphate molecules (9). PPA2 plays an important biological function in many diseases, but whether it plays a corresponding biological function in cancer? We found that its expression level can affect the prognosis of Kidney renal clear cell carcinoma (KIRC) (10). In other cancers, there are few studies on its biological function or its prognostic value as a tumor marker. Thus, this study aimed to investigate the prognostic value and diverse molecular biological functions of PPA2 in various types of cancer. This exploration encompassed examining molecular properties and pathways of interaction, as well as roles in tumor stemness and immune-related functions. The overall objective was to identify new avenues for research in prognosis prediction, critical pathway targeting, and immunotherapy across different types of cancer.

## Methods

### Analysis of gene expression in different cancers

We downloaded the standardized pan-cancer dataset from the UCSC (https://xenabrowser.net/) database: TCGA TARGET GTEx (PANCAN, N=19131, G=60499). Further, we extracted PPA2 gene expression data in each sample and excluded the cancer species with less than 3 samples in a single cancer species. Expression data for 34 different tumor types were obtained. The R package “limma”, “ggplot2” and “ggpubr” were used to compare the expression of PPA2.

In addition, the GEPIA (Gene Expression Profiling Interactive Analysis) web database were used to further validate the transcriptome expression levels of PPA2 in specific cancers (11); and the HPA (Human Protein Atlas) online website was used to explore the protein levels and subcellular location of PPA2 expression. The images of protein immunohistochemistry and immunofluorescence were downloaded here.

### Prognostic analysis of genes in different cancers

After standardized data processing, we obtained follow-up data from the public data set in UCSC as a supplement and excluded samples with a follow-up time of less than 1 month. In addition, Data transformation does not take the log value. Finally, cancer species with samples less than 10 in a single cancer species were also excluded. The result was 44 cancer species. The R package used is the Coxph function of survival (version 3.2–7), and the Cox proportional hazards regression model is established. The index we observed is the total survival. Disease-specific survival, Disease-free interval, Progression-free interval.

### Genetic alteration analysis

cBioPortal was used to collect the alteration frequency, mutation type, mutation site information, and three-dimensional (3D) structure of candidate proteins in all TCGA tumors (12).

### The single-cell analysis

CancerSEA (http://biocc.hrbmu.edu.cn/CancerSEA/home.jsp) is used to comprehensively explore the functional states of cancer cells at the single-cell level (13). The correlation data between PPA2 expression and different tumor functions based on single-cell sequencing data were analyzed. In addition, the HPA (Human Protein Atlas) database is also used to supplement single-cell analysis, particularly in breast tissue.

### The protein-protein interaction analysis of PPA2

BioGRID website aims to explore the protein-protein interaction network (14). GEPIA2.0 was used to acquire the top 100 PPA2-correlated genes from all TCGA tumors and normal tissues. Then we performed a pairwise gene-gene Pearson correlation analysis between PPA2 and the selected genes. Meanwhile, the Timer database was used to analyze the association between PPA2 and related genes.

### The analysis of modification assay and promoter methylation for PPA2

m6A(N6-methyladenosine), m1A(N1-methyladenosine), and m5C(5-methylcytosine) are the most common forms of RNA methylation modification. We extracted the expression data of the PPA2 gene and 44 marker genes of Class III RNA modifications [m1A (10), m5C (13), m6A (21)] genes in each sample, and analyzed the RNA modifications of target genes in multiple tumors. In addition, the Promoter methylation levels of PPA2 in various tumors were explored by UALCAN.

### Analysis of gene expression levels and genomic heterogeneity

Genomic heterogeneity: we analysed the TMB (Tumor mutation burden)、MSI (Microsatellite instability)、MATH (Mutant-allele tumor heterogeneity)、NEO (Neoantigen) function using R software package maftools (version 2.8.05) to calculate the results of each tumor.

### Objective to analyze the immune-correlation of genes in multiple tumors

Analysis of immune regulatory genes: we extracted PPA2 and 150 markers genes of five immune pathways [chemokine (41), receptor (18), MHC (21), Immuno-inhibitor (24) and Immuno-stimulator (46)] expression data in each sample.

Immune checkpoint gene analysis: We extracted The PPA2 gene and 60 genes of two types of Immune checkpoint pathways [Inhibitory (24) and Stimulatory (36)] marker gene expression data in each sample.

Immune cell analysis: Next, we use tumor samples from different tumor types, For the six types of immune cell infiltration scores, Pearson’s correlation between gene and immune cell infiltration scores in each tumor was calculated using the corr.test function of R package psych (version 2.1.6) coefficient, to determine the immune-infiltration score of significant correlation.

Immuno-infiltration analysis: Finally, Pearson’s correlation coefficient of PPA2 gene and immune-infiltration score in each tumor was calculated using the corr.test function of R package psych (version 2.1.6) to determine the immune-infiltration score that was significantly correlated.

### Cell culture and transfection

The breast cancer cell lines MCF-7 and MDA-MB-231 were grown in DMEM medium with Penicillin-Streptomycin Solution and 10% foetal bovine serum. The PPA2 knockdown lentivirus were purchased from OBiO Technology (Shanghai, China).

### Western blotting

Protein expression was analyzed by using the western blot assay, and the experimental procedures for western blotting was outlined in this research (15). The following antibodies were used to assay: the anti-PPA2 antibody was obtained from (Proteintech), the anti-β-actin antibody was obtained from Cell Signaling Technology.

### Clone formation assay

The breast cancer cells were inoculated into the six-well plate at a density of 200 cells per well, supplemented with 3mL medium, and cultured in the incubator for 2 weeks. Then, colonies were washed with PBS and added 1mL 0.1% crystal violet to each hole, and finally took pictures and counted.

### CCK8 assay

The breast cancer cells were inoculated in 96-well plates, the inoculated amount was about 3000 cells/well (100μl medium), and pre-cultured in an incubator (37°C, 5% CO2) for 12 hours. The experimental group and the control group were added reagents at the same time point (12h,24h,36h,48h,60h) according to the instructions. After 2h reaction, the absorbance (OD value) of 450nm wavelength was detected by enzyme-labeled instrument.

### Migration and invasion assay

Migration assay: The breast cancer cells were inoculated in a six-well plate (t 1×106 cells/well per well), then scratched with a 200 μl pipette tip and rinsed with phosphate buffer. Then, the cells were cultured in serum-free medium. The images (10x microscopic field) were recorded at 0 and 48 h respectively, and the scratch healing rate of different groups was calculated by ImageJ software.

Invasion assay: For the invasion assay, the chamber was coated with Matrigel, and breast cancer cells with 300 μL cell suspension (serum-free DMEM medium) werr added to the upper transwell chamber, and 500 μL medium containing 20% fetal bovine serum was added to the lower chamber, respectively. After incubating at 37°C for 48 h, gently remove those cells that did not invasion, the upper chamber was observed under electron microscope (10x field) and photographed.

### Drug susceptibility prediction related to PPA2 expression levels

We utilized the Genomics of Drug Sensitivity in Cancer (GDSC) database to predict the drug sensitivity linked to PPA2 in breast cancer, and the IC50 value was used to reflect the degree of tumor tolerance to the drug.

### Statistical analysis

The Wilcoxon Rank Sum Test and the Kruskal-Wallis Test were used for the comparison of gene expression differences, and the prognostic significance was obtained by statistical test using the log-rank test. Spearman or Pearson correlation analysis was employed to calculate the correlation between the two groups. The above statistical analysis and visualization were performed with R software. P < 0.05 (*), P <0.01 (**), and P < 0.001 (***), were considered significant.

## Results

### The pan-cancer analysis of PPA2 expression level

**Figure 1:**
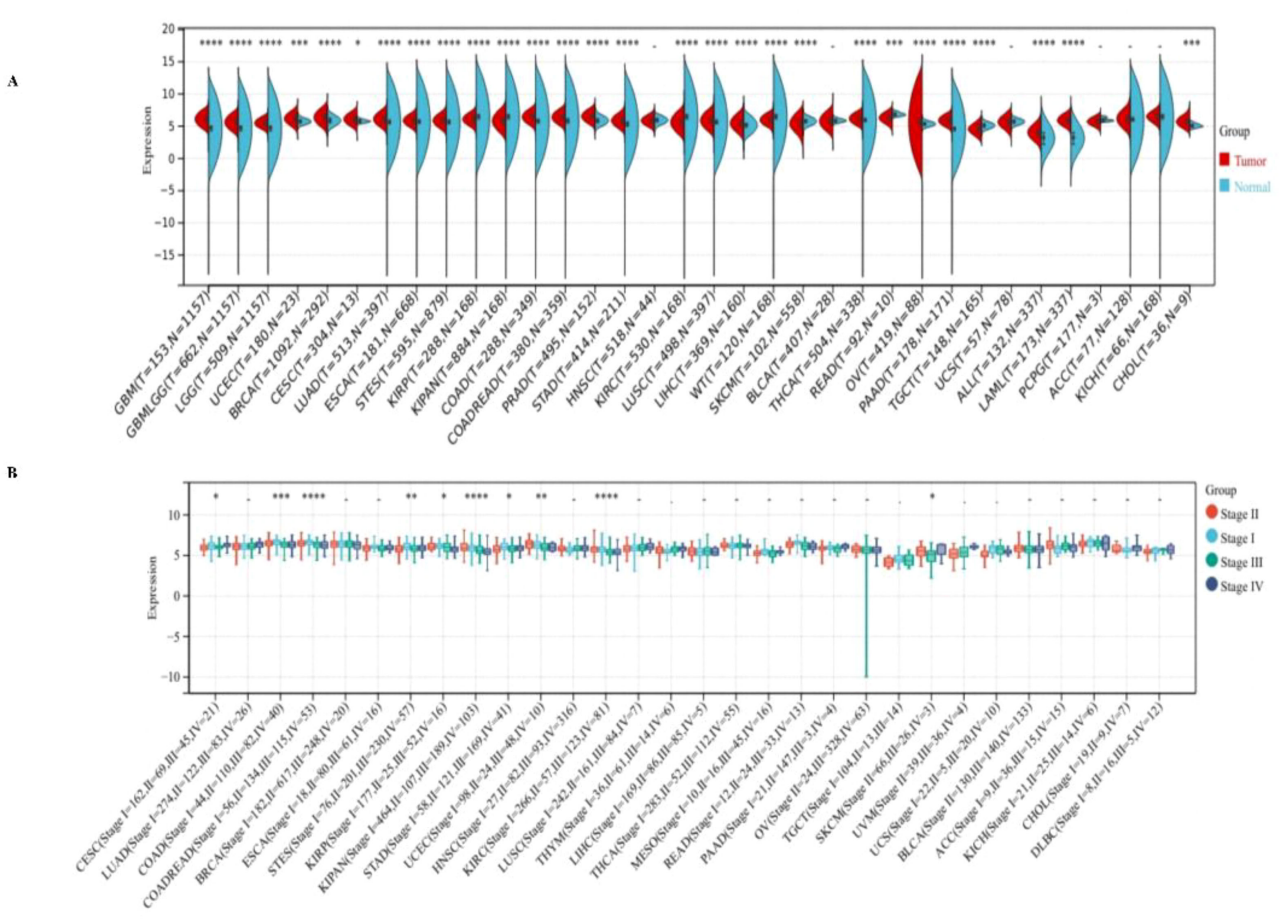
Expression levels of PPA2 in different tumors and pathological stages by TCGA + GTEx database. **(A)** Expression levels of PPPA2 in different tumors vs corresponding controls were analyzed **(B)** Analysis of the expression difference of the PPA2 gene in samples of different clinical stages in each tumor. *P<0.05; **P<0.01; ***P<0.001; ****P<0.0001.

We observed significant up-regulation of PPA2 in 21 tumors (**Figure 1A**), these include Glioblastoma multiforme (GBM), Glioma (GBMLGG), Brain Lower Grade Glioma (LGG), Uterine Corpus Endometrial Carcinoma (UCEC), Breast invasive carcinoma (BRCA), Cervical squamous cell carcinoma and endocervical adenocarcinoma (CESC), Lung adenocarcinoma (LUAD), Esophageal carcinoma (ESCA), Stomach and Esophageal carcinoma (STES) Colon adenocarcinoma (COAD), Colon adenocarcinoma/Rectum adenocarcinoma Esophageal carcinoma (COADREAD), Prostate adenocarcinoma (PRAD), Stomach adenocarcinoma (STAD), Lung squamous cell carcinoma (LUSC), Liver hepatocellular carcinoma (LIHC), Thyroid carcinoma (THCA), Ovarian serous cystadenocarcinoma (OV), Pancreatic adenocarcinoma (PAAD), Acute Lymphoblastic Leukemia (ALL), Acute Myeloid Leukemia (LAML), Cholangiocarcinoma (CHOL) (all p<0.05), we observed significant downregulation of PPA2 in 7 tumors (**Figure 1A**), such as Kidney renal papillary cell carcinoma (KIRP), Pan-kidney cohort ((KICH+KIRC+KIRP)KIPAN), Kidney renal clear cell carcinoma (KIRC), High-Risk Wilms Tumor (WT), Skin Cutaneous Melanoma (SKCM), Rectum adenocarcinoma (READ), Testicular Germ Cell Tumors (TGCT) (all p<0.05). These results indicated that PPA2 is typically highly expressed in multiple types of tumors.

Additionally, we also analyzed the expression of PPA2 in different clinical stage samples in each tumor (**Figure 1B**), and significant differences were observed in 10 tumors, such as CESC (Stage I=162, II=69, III=45, IV=21) (p=0.03), COAD (Stage I=44, II=110, III=82, IV=40) (p=8.4e-4), COADREAD (Stage I=56, II=134, III=115, IV=53) (p=4.0e-5), STES (Stage I=76, II=201, III=230, IV=57) (p=4.0e-3), KIRP (Stage I=177, II=25, III=52, IV=16) (p=0.02), KIPAN (Stage I=464, II=107, III=189, IV=103) (p=1.0e-10), STAD (Stage I=58, II=121, III=169, IV=41) (p=0.02), UCEC (Stage I = 98, II=24, III = 48, IV=10) (p=5.4e-3), KIRC (Stage I = 266, II = 57, = 123, III, IV=81) (p=1.8e-6), SKCM ((Stage II=66, III=26, IV=3)(p=0.03).

**Figure 2:**
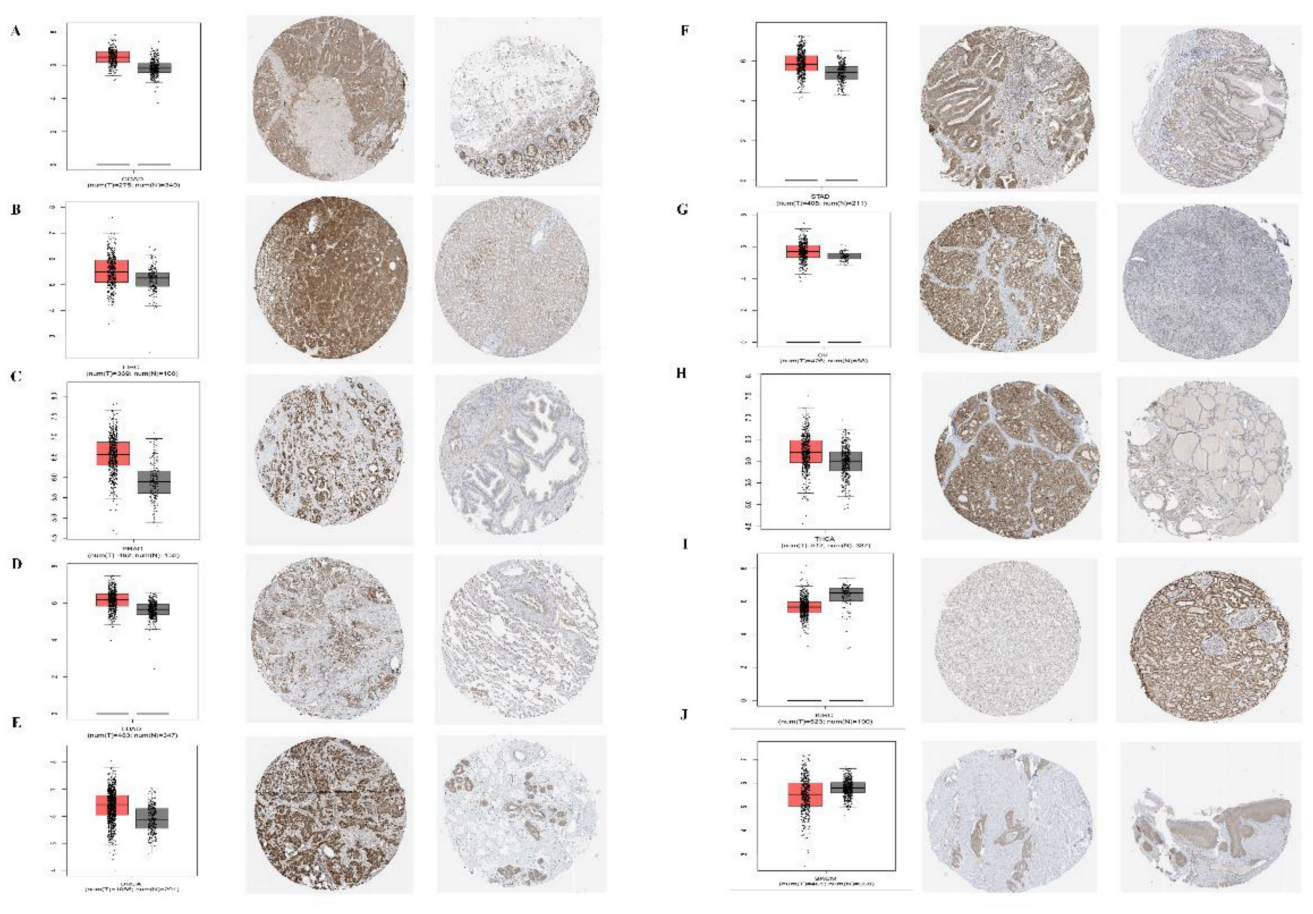
Expression levels of PPA2 in different tumors and paired normal tissues were compared in transcriptome and protein levels. **(A–H)** PPA2 expression was significantly higher in colon adenocarcinoma (COAD), liver hepatocellular carcinoma (LIHC), Prostate adenocarcinoma (PRAD), Lung adenocarcinoma (LUAD), breast invasive carcinoma (BRCA), Stomach adenocarcinoma (STAD), Ovarian serous cystadenocarcinoma (OV), Thyroid carcinoma (THCA); **(I, J)** PPA2 expression was significantly lower in Kidney renal clear cell carcinoma (KIRC), Skin Cutaneous Melanoma (SKCM).

In addition, for the accuracy of the analysis, we assessed the common tumor expression of PPA2 in other databases, and we found that the results of the analysis were consistent with the above conclusions, especially for the analysis of the protein expression level of PPA2 in a variety of tumors (**Figure 2**), which further confirmed the reliability of our conclusions. At last, we found that PPA2 expression is mainly distributed in mitochondria in the subcellular structure (**Supplementary Figure 1A**), and the results of immunofluorescence in different tumor cells also confirmed this conclusion (**Supplementary Figures 1B–D**).

### Prognostic analysis

**Figure 3:**
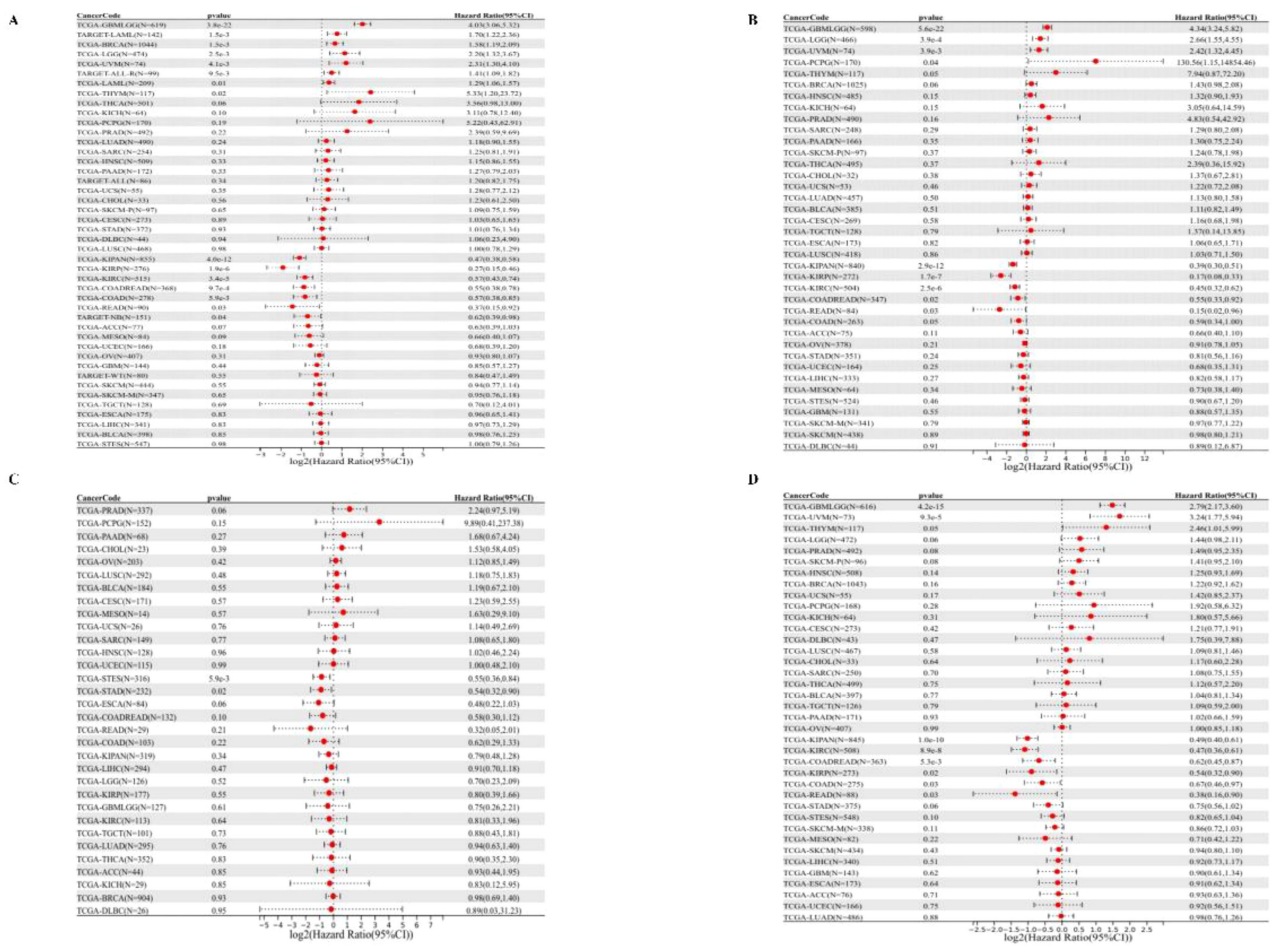
Univariate COX regression analysis of PPA2 with prognostic indicators in pan-cancer. **(A)** Correlation between PPA2 expression and OS; **(B)**, Correlation between PPA2 expression and DSS. **(C)** Correlation between PPA2 expression and DFI; **(D)** Correlation between PPA2 expression and DFI.

We started by analyzing the relationship between gene expression and OS in each tumor, and the results showed that the high expression of PPA2 in eight tumors had a poor prognosis (**Figure 3A**), including TCGA – GBMLGG, p= 3.8e-22), TCGA- LGG (p=2.5e-3), TARGET - LAML (p=1.5e-3), TCGA - BRCA (p =1.5e-3), TCGA THYM (p=0.02), TCGA UVM (p = 4.1e-3), TCGA - LAML (p = 0.01), TARGET - ALL - R (p = 9.5e-3).In addition, the results (**Figure 3A**) suggested that low PPA2 expression in seven tumors had a poor prognosis:(TCGA - KIRP (P=1.9e-6), TCGA - KIPAN (p = 4.0e-12), TCGA COAD (p=5.9e-3), TCGA - COADRE AD (p=9.7e-4), TCGA KIRC (p=3.4e-5), TARGET - NB (p=0.04), TCGA - READ (p=0.03). Further, we performed survival curves for cancers with statistically different OS levels of high PPA2 expression with a poor prognosis (**Supplementary Figure 2**). These tumors of low PPA2 expression with a poor prognosis were shown in the **Supplementary Figure 3**.

For DSS (**Figure 3B**), We found that the final observed in 4 tumor types (TCGA - GBMLGG (p=5.6e-22), TCGA LGG (p=3.9e-4), TCGA - UVM (p=3.9e-3), TCGA PCPG (p=0.04) with poor prognosis, high expression in five types of tumors (TCGA KIRP - (p=1.7e-7), TCGA - K IPAN (p=2.9e-12), TCGA COADREAD (p = 0.02), TCGA KIRC (p=2.5e-6), TCGA - Low READ(p=0.03) had a poor prognosis.

For DFI (**Figure 3C**), we found that low expression in TCGA-STES (p=5.9e-3) and TCGA-STAD (p=0.02) had poor prognosis in the two tumor types.

Finally, for PFI (**Figure 3D**), We found that in the two tumor types (TCGA - GBMLGG (p=4.2e-15), TCGA UVM (p=9.3e-5) with poor prognosis, high expression in the six tumor type (TCGA - KIRP (p=0.02), TCGA KIPAN (p=1.0 e-10), TCGA COAD (p=0.03), TCGA - COADREAD (p=5.3e-3), TCGA KIRC (p=8.9e-8), TCGA - READ (p=0.03) and low expression have poor prognosis.

### PPA2 mutation in various tumors

**Figure 4:**
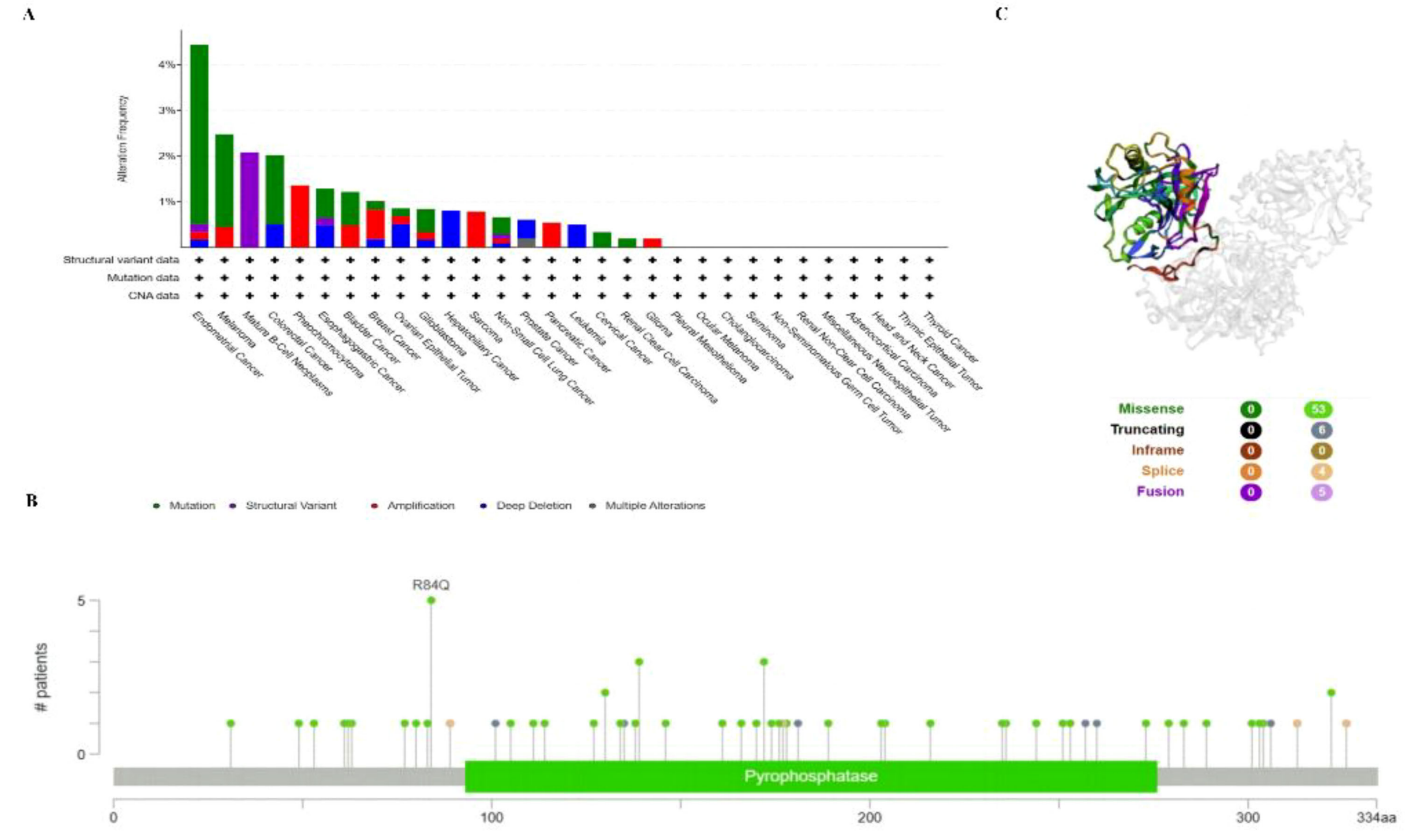
PPA2 gene mutation in various cancers. cBioPortal was used to display the alteration frequency of different mutation types **(A)** and mutation sites **(B)** of PPA2 in pan-cancer. **(C)** The R84Q alteration mutation site was shown in the 3D protein structure of PPA2.

To investigate the gene mutation of PPA2 in various tumors, we analyzed its mutation status through the cBioPortal platform based on TCGA data. For instance, Pan-cancer analysis (**Figure 4A**) suggested the highest gene alter in Endometrial Cancer (4.44%); Mature B-cell neoplasms suggested the highest “Structural Variant” incidence (2.08%); Pheochromocytoma suggested the highest “Amplification” incidence (1.36%). In addition, the result displayed the R84Q alteration in the structure of the PPA2 protein **Figure 4B**. Finally, we found that missense and truncating were the predominant mutation styles in PPA2, and the mutation site can be viewed in 3D structure which is shown in **Figure 4C**.

### Protein-protein interaction analysis

**Figure 5:**
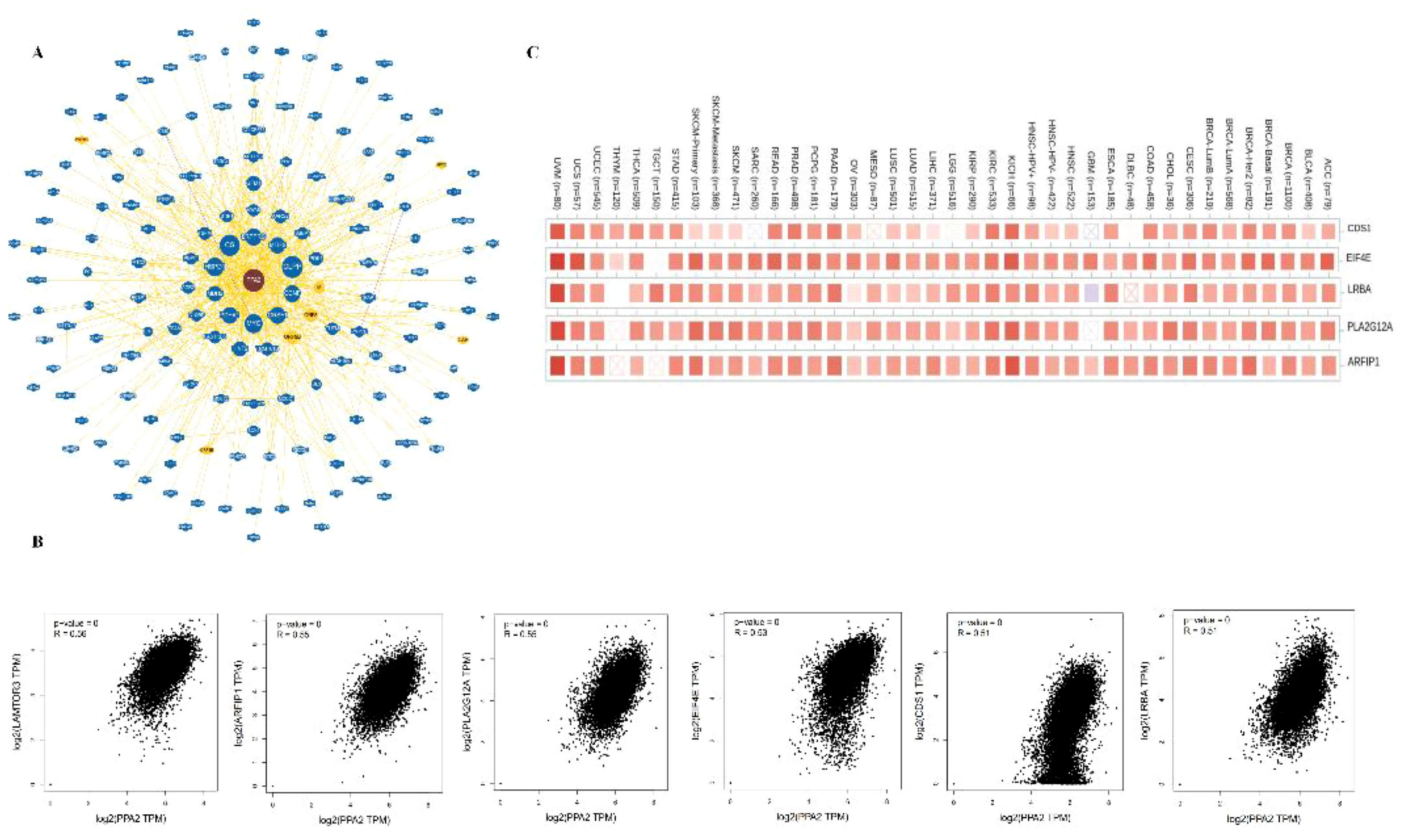
The Protein-protein interaction analysis of PPA2. **(A)** PPA2-related genes were obtained from the BioGRID web tool **(B)** GEPIA2.0 showed positive correlations between PPA2 and six genes (LAMTOR3, CDS1, EIF4E, LRBA, PLA2G12A, and ARFIP1). **(C)** The heatmap indicated that PPA2 expression was positively correlated with these genes (CDS1, EIF4E, LRBA, PLA2G12A, and ARFIP1) in pan-cancer.

In addition, the potential interaction of PPA2 molecules is demonstrated from the BioGRID web tool. Firstly, we acquired the interacting gene networks (**Figure 5A**). Next, we acquired the top 10 PPA2-correlated genes from all TCGA tumors and normal tissues, among these genes, LAMTOR3, CDS1, EIF4E, LRBA, PLA2G12A, and ARFIP1) showed high correlations (all r>0.05,p<0.05)with PPA2 (**Figure 5B**); in addition, the Timer database showed that these genes (CDSA, EIF4E, LRBA PLA2G12A, and ARFIP1) are closely related to PPA2 in the majority of cancer types (**Figure 5C**).

### Analysis of RNA gene modification

**Figure 6:**
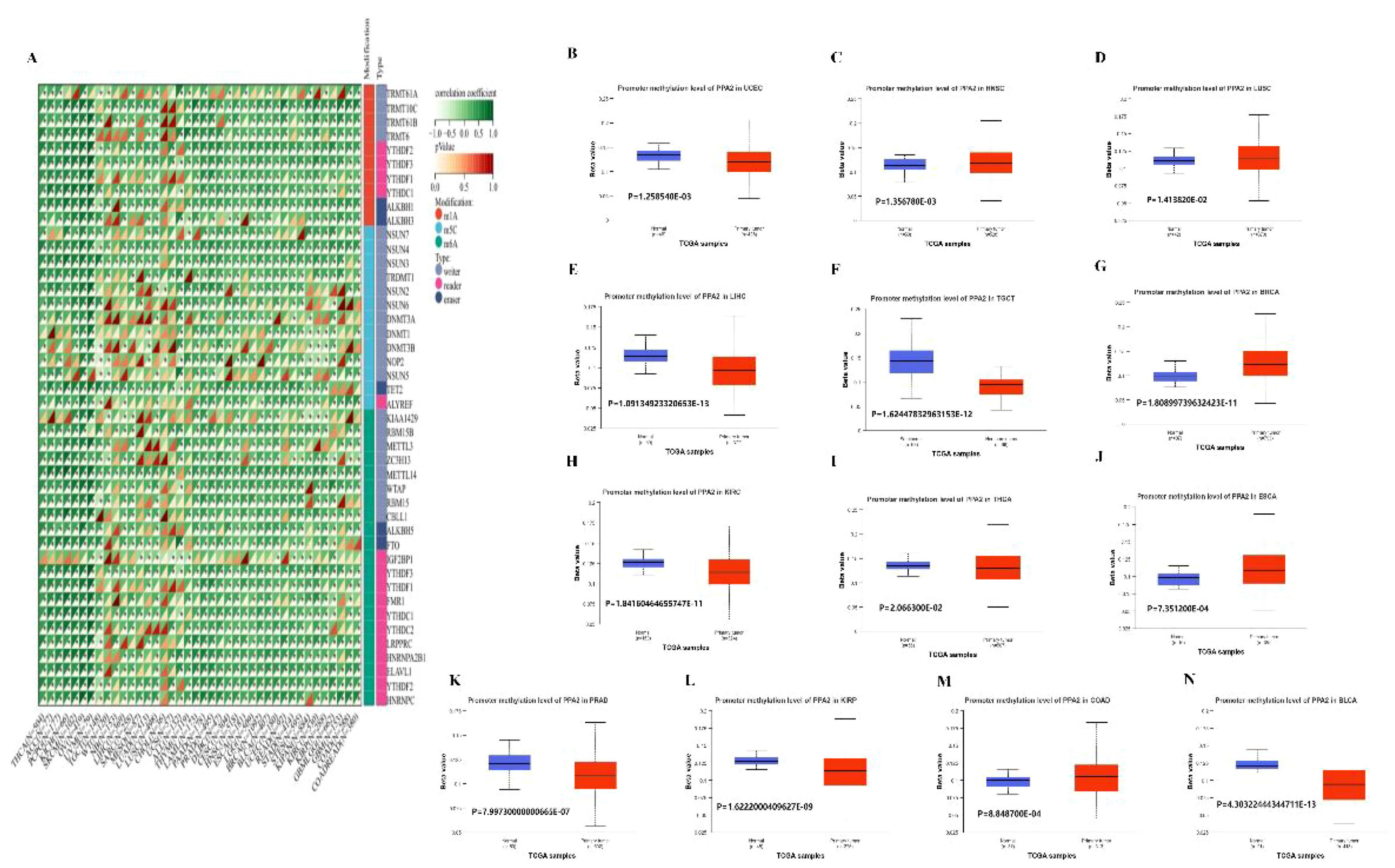
The analysis of RNA gene modification and promoter methylation levels for PPA2. **(A)** The relationship between PPA2 expression and RNA gene modification **(B–N)** The relationship between PPA2 expression and promoter methylation levels among pan-caner.

Moreover, in order to further explore the biological role of PPA2 in different tumors, we extracted the expression data of the PPA2gene and 44 marker genes of Class III RNA modification [m1A (10), m5C (13), m6A (21)] genes in each sample to investigate the relationship between PPA2 and RNA modification genes in different tumors. In these cancers, we found that PPA2 expression was significantly correlated with RNA-modifying genes among the majority of tumors. Our analysis results also displayed that promoter methylation levels of PPA2 were significantly reduced in several tumor tissues (**Figure 6A**), including UCEC (p=1.258540E-03), HNSC (p=1.356780E-03), LUSC (p=1.413820E-02), KIRP (p=1.6222000409627E-09), LIHC (p=1.09134923320653E-13), TGCT (p=1.62447832963153E-12), BRCA (p=1.80899739632423E-11), KIRC (1.84160464655747E-11), THCA (p=2.066300E-02), BLCA (p=4.30322444344711E-13), ESCA (p=7.351200E-04), PRAD (p=7.99730000000665E-07), COAD (p=8.848700E-04).

In addition, many tumors exhibit significant differences in PPA2 promoter methylation levels between tumor and normal tissues (**Figures 6B–N**), including HNSC (p=1.356780E-03), LUSC (p=1.413820E-02), BRCA (p=1.80899739632423E-11), ESCA (p=7.351200E-04), COAD (p=8.848700E-04), TGCT (p=1.62447832963153E-12), UCEC (p=1.258540E-03), LIHC (p=1.09134923320653E-13), KIRC (p=1.84160464655747E-11), THCA (p=2.066300E-02), PRAD (p=7.99730000000665E-07), KIRP (p=1.6222000409627E-09), BLCA (p=4.30322444344711E-13).

### Gene expression levels were analyzed in relation to Gene heterogeneity

**Figure 7:**
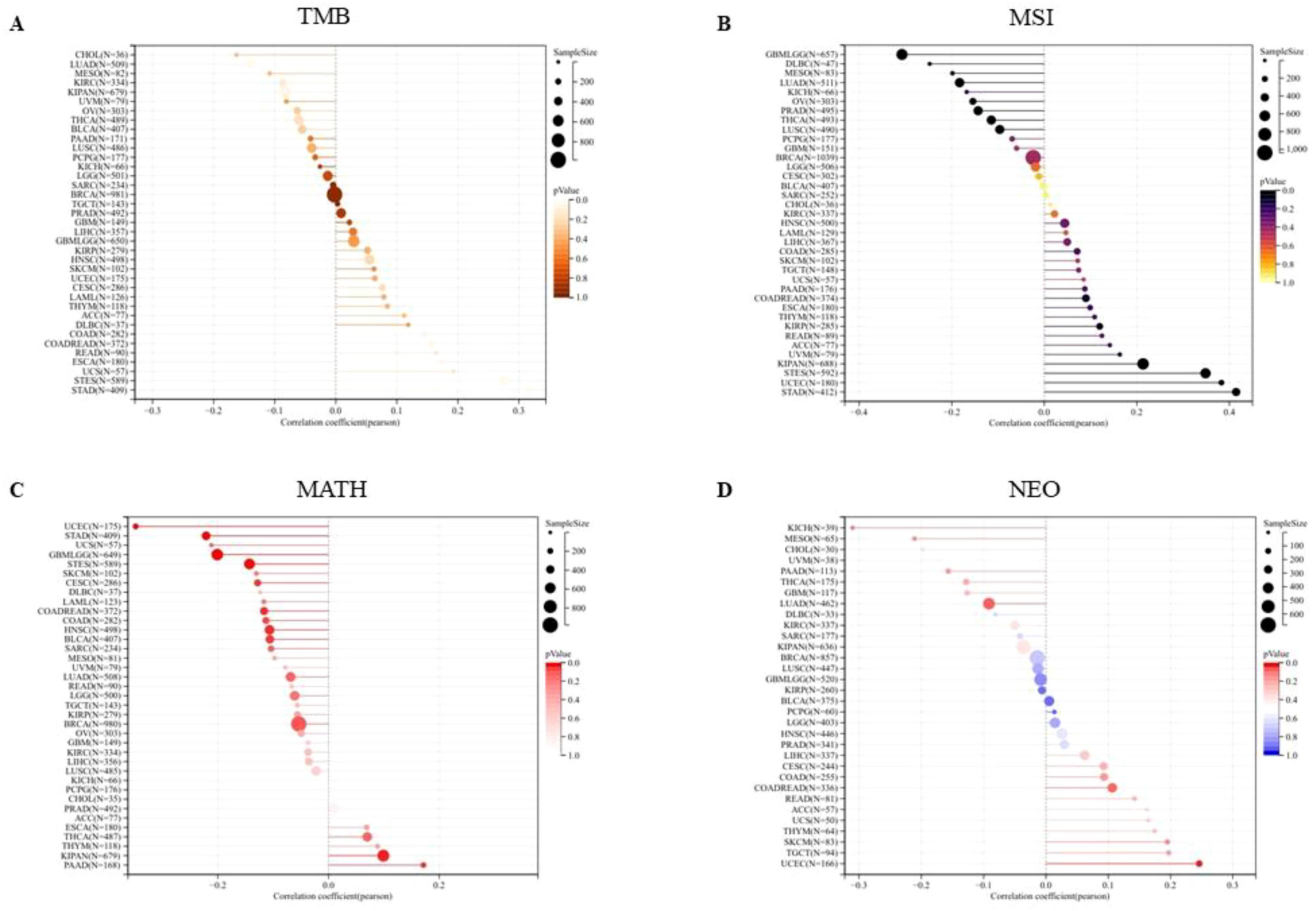
The association between PPA2 expression levels and gene heterogeneity. **(A)** Correlation between PPA2 expression and TMB; **(B)** Correlation between PPA2 expression and MSI **(C)** Correlation between PPA2 expression and MATH; **(D)** Correlation between PPA2 expression and NEO.

We first analyzed the association between PPA2 expression levels and Tumor Mutation Burden (TMB). The results indicated that there was a significant positive correlation among five tumors (**Figure 7A**), such as COAD (N=282) (R=0.144318391555262, P=0.0152888308912382), COADREAD (N=372) (R=0.156501798188948, P=0.00246983956445935), ESCA (N=180) (R=0.173012148283644, P=0.0202020885505813), STES (N=589) (R=0.275197953683056, P=1.07309945917818e-11), STAD (N=409) (R=0.315502654670929, P=6.63846553762865e-11), there was a significant negative correlation between the two tumors. For example, LUAD (N=509) (R=-0.140749683744781, P=0.00147088281564788), KIPAN (N=679) (R=0.0826126229519112, P=0.0313656323395094); Next, we integrated and analyzed the Microsatellite Instability(MSI) and gene expression data of PPA2 in the samples (**Figure 7B**), and observed that PPA2 was significantly correlated in 11 tumors, including 5 tumors (STES、KIPAN、STAD、UCEC、KIRP) with significantly positive correlation and 6 tumors(GBMLGG、LUAD、PRAD、LUSC、THCA、OV) with significant negative correlation(all p<0.05).

In addition, the mutant-allele tumor heterogeneity (MATH) and the Neoantigen (NEO) analysis results also indicated that PPA2 expression levels were associated with them in many tumors (**Figures 7C, D**).

### Immuno-correlation and Immune score analysis

**Figure 8:**
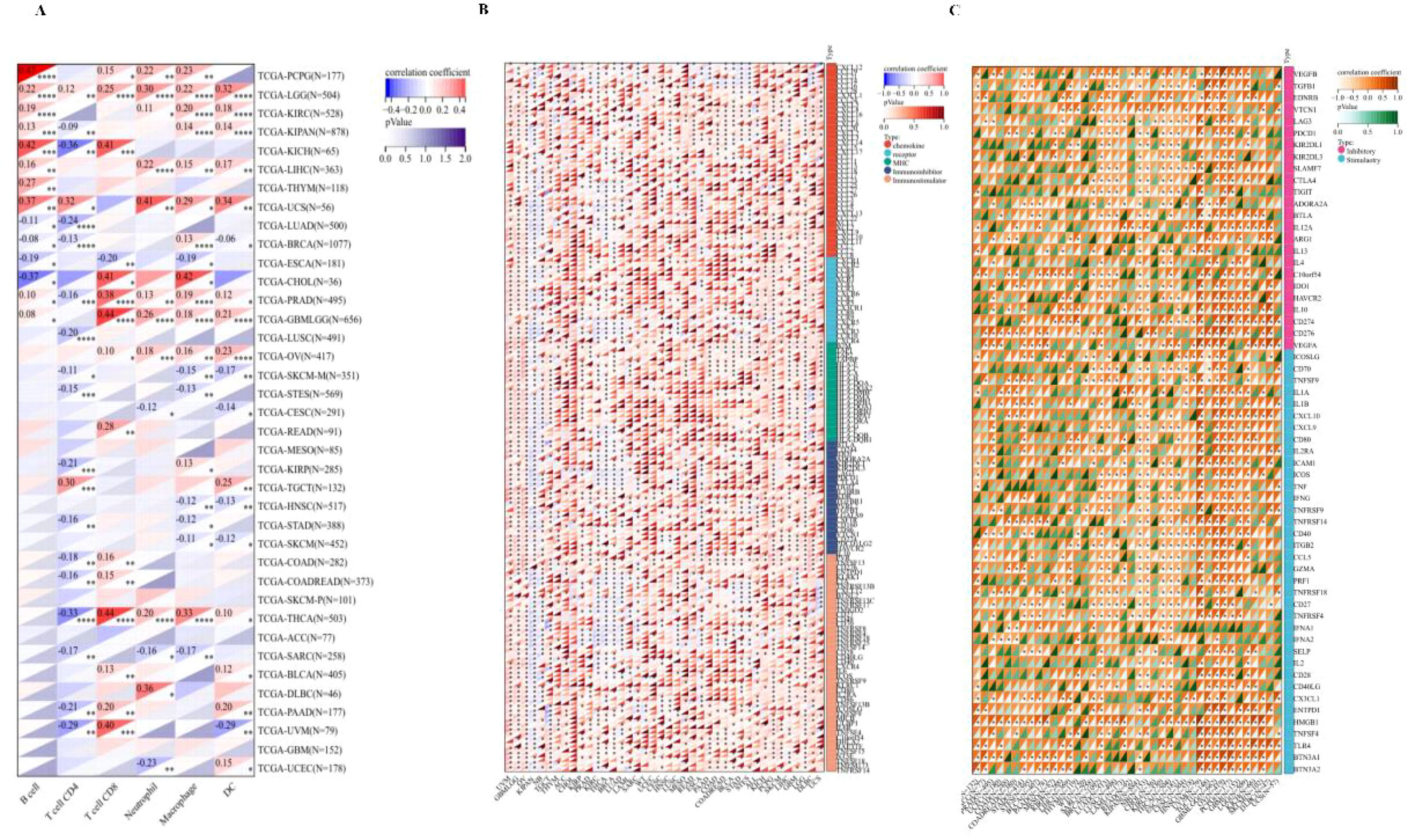
The Immuno-correlation analysis for PPA2. The immuno-correlation cell infiltration analysis **(A)** with PPA2 among different tumors. The immune checkpoint pathway analysis **(B)** and the marker genes of five immune pathways **(C)** for PPA2.

We evaluated the B cell, T cell CD4, T cell CD8, Neutrophil, Macrophage, and DC infiltration scores of each patient in each tumor based on gene expression and we obtained six types of immune cell infiltration scores for 9406 tumor samples from 38 tumor types. Finally, we observed that there were significant changes in 34 cancer species (**Figure 8A**), including (TCGA-BLCA(N=405), TCGA-BRCA(N=1077), TCGA-CESC(N=291), TCGA-CHOL(N=36), TCGA-COAD(N=282), TCGA-COADREAD(N=373),TCGA-DLBC(N=46),TCGA-ESCA(N=181), TCGA-GBMLGG(N=656), TCGA-HNSC(N=517), TCGA-KICH(N=65), TCGA-KIPAN(N=878), TCGA-KIRC(N=528), TCGA-KIRP(N=285), TCGA-LGG(N=504), TCGA-LIHC(N=363), TCGA-LUAD(N=500), TCGA-LUSC(N=491), TCGA-OV(N=417), TCGA-PAAD(N=177), TCG A-PCPG(N=177), TCGA-PRAD(N=495), TCGA-READ(N=91), TCGA-SARC(N=258), TCGA-SKCM-M(N=351), TCGA-SKCM(N=452), TCGA-STAD(N=388), TCG Expression and immunity of this gene in A-STES(N=569), TCGA-TGCT(N=132), TCGA-THCA(N=503), TCGA-THYM(N=118), TCGA-UCEC(N=178), TCGA-UCS(N=56), TCGA-UVM(N=79)) There was significant correlation between epidemic infiltration. Notably, although PPA2 significantly contributed to the infiltration of immune cells in most tumors, this relationship was not prominent in GBM, SKCM-P, and a few tumor cancer types.

We further extracted the PPA2 gene and 60 genes of two types of immune checkpoint pathways (**Figure 8B**), including Inhibitory and Stimulatory. Meanwhile, we calculated Pearson correlations between PPA2 and the marker genes of five immune pathways (**Figure 8C**), including chemokine, receptor, MHC, Immuno-inhibitor and Immuno-stimulator. It was found that these immune-related pathways were strongly correlated with PPA2 expression levels in most tumor types. Meanwhile, these immune checkpoint pathway genes provide ideas and evidence for future molecular pathway studies.

Furthermore, we also analyzed the immune-infiltration scores of 10,180 tumor samples from a total of 44 tumor types. Finally, we observed that the expression of this gene was significantly correlated with immune infiltration in 22 cancer species (**Supplementary Figure 4**), of which 2 tumors were significantly positively correlated, such as: TCGA-GBMLGG (N=656, R=0.35, P=1.5e-20)、TCGA-LGG(N=504, R=0.21, P=2.3e-6), There were 20 tumors significant negative correlations, such as TCGA-UCEC (N=178, R=-0.21, P=5.2e-3),TCGA-BRCA (N=1077, R=-0.24, P=3.7e-16),TCGA-CESC (N=291, R=-0.23,P=9.9e-5),TCGA-LUAD (N=500, R=-0.21,P=3.0e-6),TCGA-ESCA (N=181,R=-0.36, P=7.0e-7),TCGA-STES (N=569,R=-0.17, P=5.5e-5),TCGA-SARC (N=258, R=-0.16,P=0.01),TCGA-KIPAN(N=878,R=-0.42,P=5.2e-39),TCGA-PRAD(N=495,R=-0.19,P=1.3e-5),TCGA-HNSC (N=517,R=-0.18, P=2.9e-5),TCGA-KIRC (N=528,R=-0.28, P=6.7e-11),TCGA-LUSC (N=491,R=-0.16, P=3.1e-4),TCGA-SKCM-P (N=101,R=-0.28, P=4.5e-3),TCGA-SKCM (N=452,R=-0.21, P=5.8e-6),TCGA-BLCA (N=405,R=-0.12, P=0.02),TCGA-SKCM-M (N=351,R=-0.23, P=1.7e-5),TCGA-THCA (N=503,R=-0.36, P=1.1e-16),TARGET-NB (N=153,R=-0.26, P=1.4e-3),TCGA-PAAD (N=177,R=-0.25,P=8.7e-4),TCGA-ACC (N=77,R=-0.34,P=2.3e-3).

### The exploration of PPA2 gene in single-cell field

**Figure 9:**
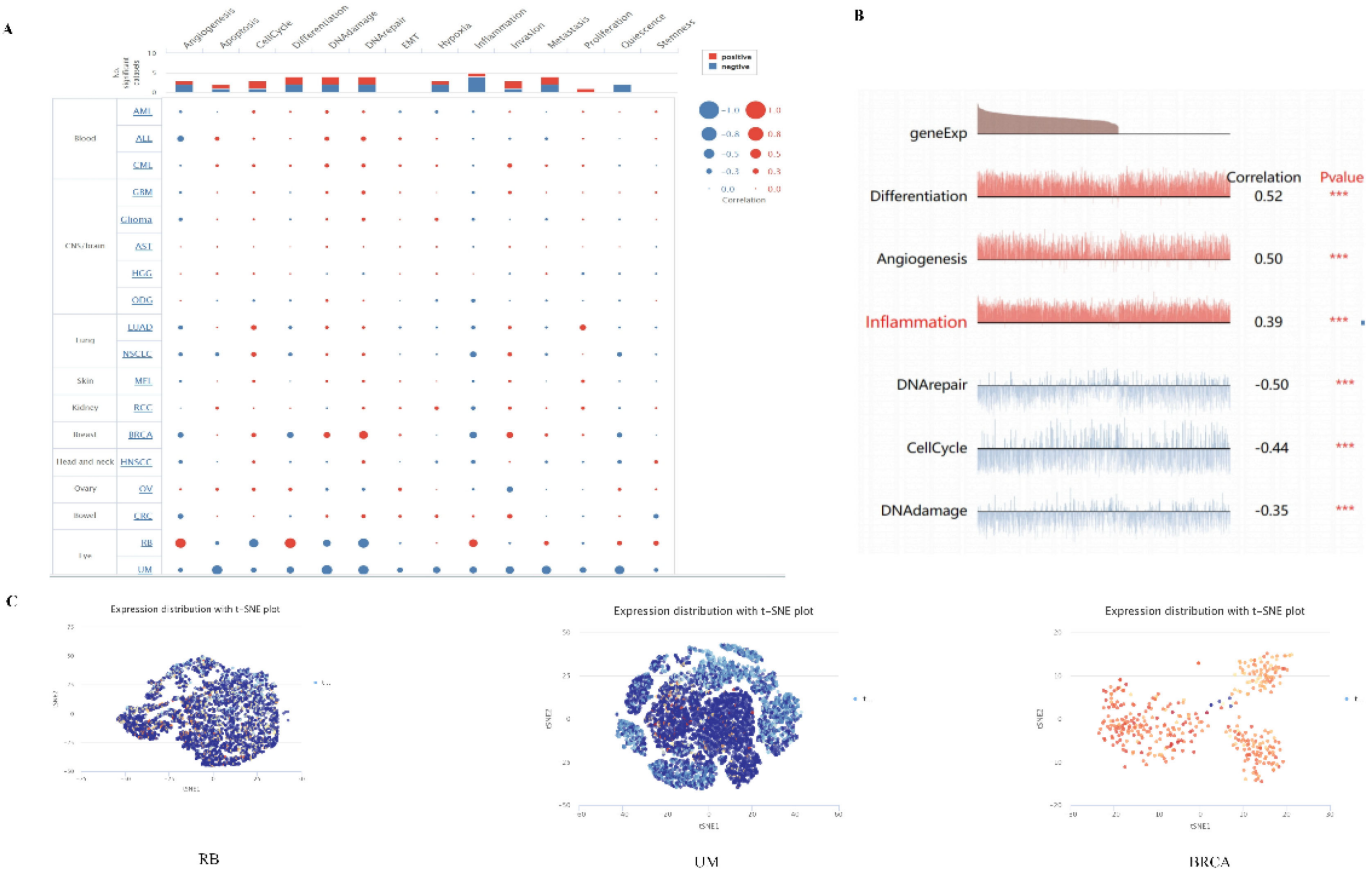
The expression levels of PPA2 at single-cell levels. **(A, B)** The relationship between PPA2 expression and different functional states in tumors was explored by the CancerSEA tool. ***p < 0.001. **(C)** PPA2 expression profiles were shown at single cells from RB, UM and BRCA by T-SNE diagram.

Single-cell transcriptome sequencing is a key technique for analyzing the underlying functions of candidate molecules at single-cell levels. We have observed that PPA2 is involved in various biological processes in a variety of tumors (**Figure 9A**). For instance, such as retinoblastoma (RB), PPA2 is positively correlated with anti-angiogenesis, inflammation, and differentiation; it is negatively correlated with DNA repair, cell cycle, and DNA damage (**Figure 9B**). In addition, the results of PPA2 expression profiles were shown at single cell levels from RB, UM, and BRCA by T-SNE diagram (**Figure 9C**).

The results showed the PPA2 expression from high to low in different cells (**Supplementary Figure 5A**). The highest expression of PPA2 was in Proximal tubular cells, and the lowest expression of PPA2 cell was in Spermatogonia. Furthermore, breast tissue was the focus of our attention, and we found that each cell type was divided into different cell clusters according to different levels of PPA2 nTPM expression (**Supplementary Figure 5B**). In addition, we explored the cell type markers, the results (**Supplementary Figure 5C**). indicated that clusters of cells ranked were breast glandular cells c-4 and breast glandular cells c-16 compared with the other cells.

### PPA2 promotes the proliferation of breast cancer cells

**Figure 10:**
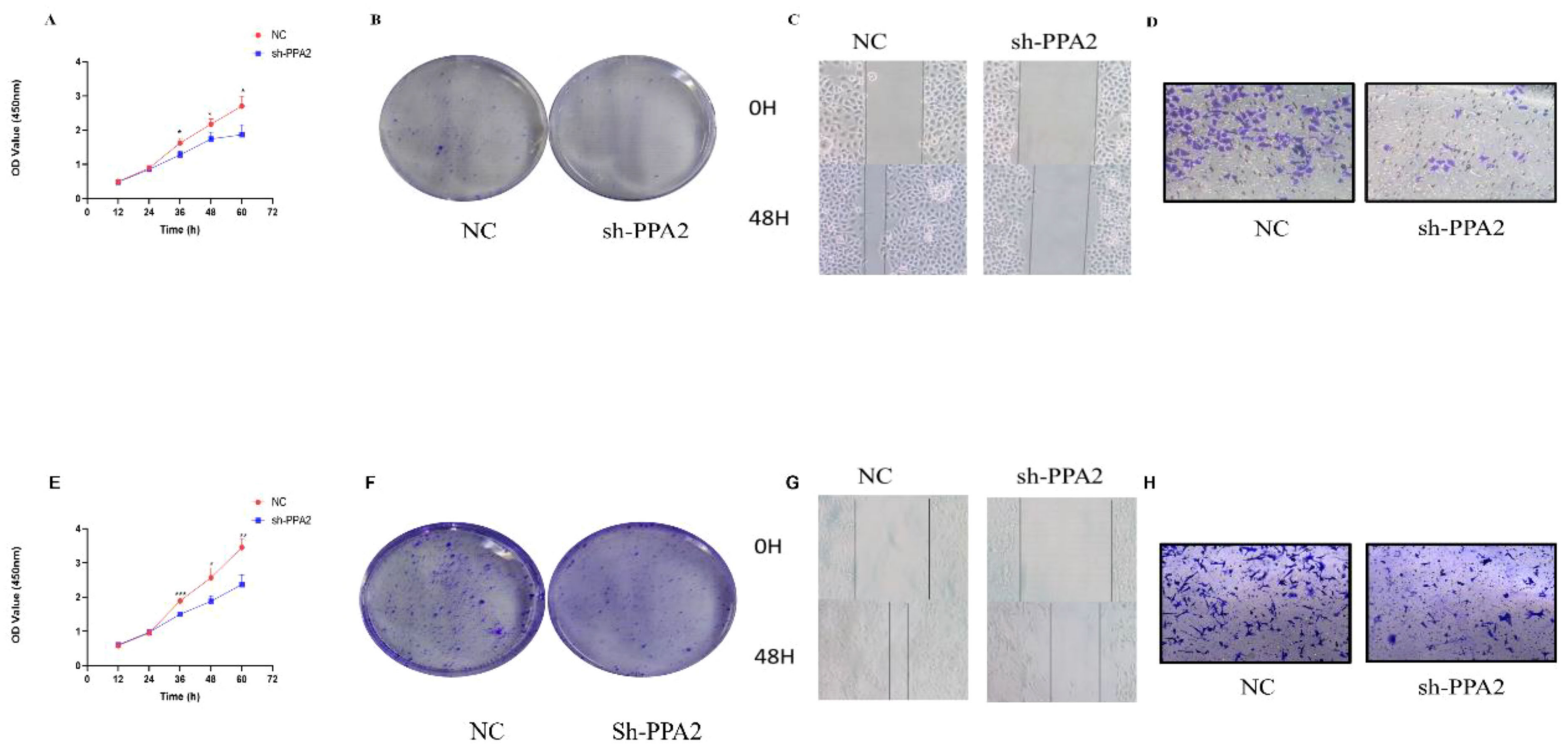
PPA2 knockdown inhibited the progression in breast tumor cells. PPA2 knockdown inhibited proliferation in MCF-7 cells **(A, B)** and MDA-MB-231 cells **(E, F)**; PPA2 knockdown inhibited migration and invasion in MCF-7 cells **(C, D)** and MDA-MB-231 cells **(G, H)**.

We first verified the PPA2 knockdown efficiency (**Supplementary Figures 6A, E**), and then selected the most efficient target sequence for subsequent validation. CCK8 and clone formation assay were applied to explore the proliferative ability of the breast cancer cells. The results showed that the number of cells decreased significantly when MCF-7 cells were treated with PPA2 shRNA (**Figure 10A**). Similarly, the results of the clone formation experiment showed that the number of MCF-7 cells in the sh-PPA2 group was lower than the control group (**Figure 10B**), with a statistically significant difference (**Supplementary Figure 6B**). Similarly, we found similar results in the MDA-MB-231 cells (**Figures 10E, F**), and the number of MDA-MB-231cells in the sh-PPA2 group was significantly lower than that in the control group (**Supplementary Figure 6F**). These results demonstrated that PPA2 may promote the proliferation of breast cancer cells.

### PPA2 promotes migration and invasion of breast cancer cells

Wound Healing and Transwell assay were applied to explore the migration and invasion ability of breast cancer cells respectively. The migration and invasion ability of MCF-7 cells treated with PPA2 shRNA was significantly weaker than control group (**Figures 10C, D**), with a statistically significant difference (**Supplementary Figures 6C, D**). Similarly, the results of the migration and invasion ability were also significantly inhibited in the MDA-MB-231cells with PPA2 shRNA (**Figures 10G, H**), with a statistically significant difference (**Supplementary Figures 6G, H**). Which confirmed that knocking down PPA2 expression levels inhibited tumor progression.

### Prediction of drug susceptibility to abnormal PPA2 expression in breast cancer

**Figure 11:**
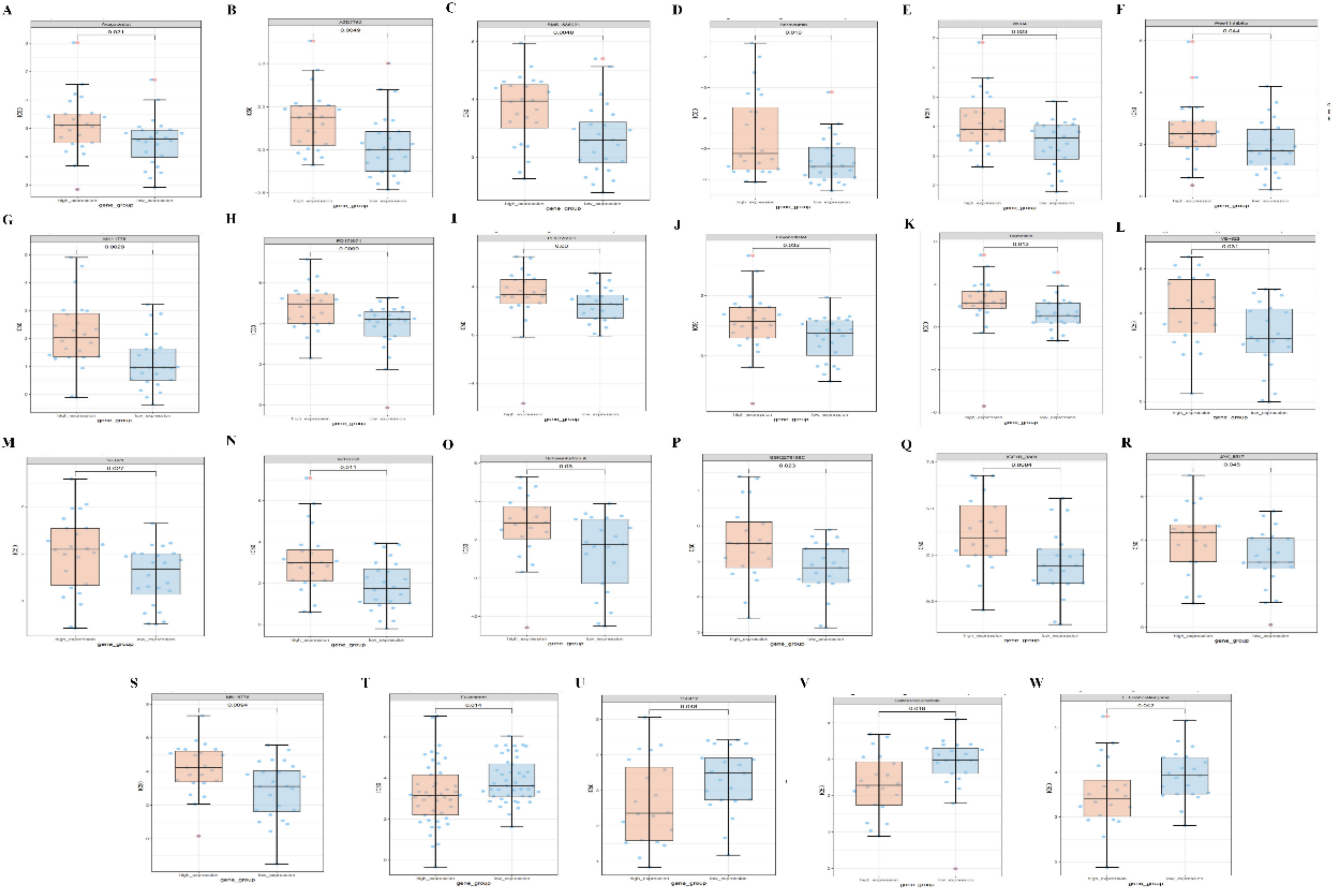
Correlations between PPA2 expression levels and different drug sensitivities. The PPA2 expression was related to drug susceptibility in breast cancer **(A–W)**.

Finally, we predict the level of PPA2 expression and the sensitivity of different compounds or drugs. The aim is to conduct clinical translational studies of drugs among people with abnormal PPA2 expression. We found that the high expression of PPA2 is associated with increased IC50 of many drugs such as Avagacestat (**Figures 11A–S**). On the contrary, the low expression of PPA2 is associated with increased IC50 of some drugs such as Fulvestrant (**Figures 11T–W**).

## Discussion

Pan-Cancer Analysis is a very valuable part of tumor bioinformatics analysis, and many related studies have revealed the value of pan-cancer analysis in the treatment of tumors or in guiding prognosis (16–21). At present, the systemic treatment plan for cancer has entered the era of individuation and precision, and we will use different treatment plans for different patients with the same disease (22, 23). However, according to the classification and division at the molecular level such as genome, transcriptome, and proteome, the same molecules that are abnormally expressed in different diseases can also be applied to the same therapeutic measures (24, 25). For example, Trastuzumab deruxtecan (T-DXd) is a human epidermal growth factor 2 (HER2) -directed antibody-drug conjugated compound that can be used in breast and stomach cancers with high HER2 expression and in non-small cell lung cancers with HER2 mutations (26). Therefore, different patients with different diseases, using the same treatment plan, is also a kind of precision treatment. The therapeutic principle that is relied upon at this time is that different diseases may have common causative factors. Although the diseases are different, the same therapeutic measures can be used depending on the specific molecular target. Hence, this is the significance of pan-cancer analysis, although the tumor is different, but finds the biological function of the same molecule in different tumors, which can help tumor research and patient treatment. Thus, our study aims to investigate some co-expressed pathogenic factors found in different tumors, so as to find some targets with therapeutic potential and provide research directions for the treatment of different cancers in the future.

Some studies have indicated that PPA2 may exhibit varying roles across diseases, however, there is limited research on its role in tumors, presenting significant research opportunities (27, 28). As a result, our initial discovery revealed that PPA2 expression was aberrant in various types of cancers, showing higher levels in multiple tumors than in normal tissues. In addition, in the subcellular structure, the expression of PPA2 is mainly distributed in mitochondria. The above analysis of PPA2 expression or distribution provided us with a preliminary understanding of the status of PPA2 in tumors. Next, we analyzed the prognostic value of PPA2 in a variety of tumors, respectively from four different indicators of OS, DSS, DFI, and PFI. We found that the difference in the expression level of PPA2 was statistically significant with that of OS, DSS, DFI and PFI in a variety of tumors. This also suggested that PPA2 has the potential to be used as a valuable marker of prognosis in a variety of tumors. At the same time, the gene mutation status and co-expression network of PPA2 in tumors were also analyzed.

In addition, we also explored the more common internal modifications of mRNA, including N6-adenylate methylation (m6A), N1-adenylate methylation (m1A), and cytosine hydroxylation (m5C). The analysis results also confirmed that PPA2 is closely associated with RNA methylation-related genes in a variety of tumors. Next, we analyzed some biological characteristics (TMB, MSI, MATH, NEO) of PPA2 in different tumors. These indicators are considered to be predictors of tumor immunotherapy response (29–31), so the analysis of the correlation between the expression level of PPA2 and these indicators is also to observe the immune value of PPA2 in the treatment of different tumors.

In addition to the biological properties mentioned above, one of the most important analyses was the relationship between PPA2 and immuno-correlation analysis. Because immune research on tumors has become a favorable weapon in tumor treatment in recent years, various reports on immunity also show important value (32, 33). We analyzed the role of PPA2 in immunity from different dimensions. First, we analyzed the immune infiltration level of PPA2 in different tumors. In our analysis of PPA2 in different tumors, we observed a correlation between PPA2 and immune infiltration in the majority of cases. About two-thirds of these tumors showed a negative correlation; Next, we investigated the role of PPA2 in tumors and a variety of immune cells (including B cell, T cell CD4, T cell CD8, Neutrophil, Macrophage, DC) and found a clear association between PPA2 and various cells. These results suggest that the expression of this gene is significantly correlated with immune infiltration. Numerous studies have shown a correlation between immune infiltration and the prognosis and treatment response in various types of human cancers (34). Specifically, in non-small cell lung cancer, tumors with robust immunotherapy efficacy are considered “hot tumors” due to their favorable immunoinfiltrating status (35, 36). Conversely, tumors categorized as “cold tumors,” which exhibit resistance to immunotherapy, are commonly identified as “immune deserts” characterized by deficient immune cell infiltration (37).

In addition, the marker genes of PPA2 and five types of immune pathways as well as immune checkpoint Pearson correlation analysis were performed. As we all know, these immunomodulators and immune checkpoints are crucial for maintaining autoimmune tolerance and regulating the duration and scope of immune response in peripheral tissues (28). One promising approach to achieving anti-cancer immunity is to block the immune checkpoint pathway, so it is necessary to analyze the immune checkpoint in different tumors for PPA2.Analysis of relevant immunomodulators and immune checkpoints is of great significance for the molecular mechanism of immune response and the development of immune checkpoint inhibitors. The results of our analysis also show that PPA2 is closely associated with various immune pathways and multiple immune checkpoints in a variety of tumors, and it has full potential to become an effective target for immunotherapy in the future.

At the same time, we conducted a series of single-cell analysis of the PPA2 gene, which is the most helpful for us to fully understand the biological role of PPA2 and reveal the role of PPA2 in breast tissue. Finally, we also conducted functional experiments in breast cancer cell lines, and found that PPA2 knockdown inhibited the proliferation, migration and invasion of tumor cells, which also suggested that PPA2 might promote the progression of breast tumor. Of course, this study also has some limitations. Currently, the verification of PPA2 expression level relies on database information. In the future, we aim to conduct IHC verification using collected samples (including in different subtypes of breast cancer tissue) to strengthen the findings. Furthermore, we have confirmed the impact of PPA2 on triple-negative and estrogen receptor-positive breast cancer cell lines. To further confirm the role of PPA2 in breast cancer, future studies recommend validating it in HER2-enriched cell lines for a more thorough and reliable analysis. In brief, the significant potential of PPA2 in the context of tumors warrants further exploration by researchers.

## Conclusion

In conclusion, we have conducted a series of analyses of PPA2 in pan-carcinoma, including its expression level in tumors and clinical prognostic value, as well as basic biological characteristics, especially its role in immunity. At last, we confirmed that PPA2 promotes the progression of breast cancer cells. These analyses confirmed that PPA2 has the potential to be used as a prognostic indicator and an immunotherapeutic target for further investigation.

## Data availability statement

The original contributions presented in the study are included in the article/**Supplementary Material**. Further inquiries can be directed to the corresponding author.

## Author contributions

HL: Conceptualization, Writing – original draft, Writing – review & editing. JZ: Investigation, Writing – original draft, Writing – review & editing. BY: Investigation, Writing – original draft, Writing – review & editing. LL: Investigation, Writing – original draft, Writing – review & editing. HX: Data curation, Writing – original draft. BW: Methodology, Writing – original draft. ZY: Supervision, Writing – review & editing. XZ: Formal analysis, Writing – review & editing.
