# Supplementary figures and images for: Multi-omics pan-cancer analysis reveals the prognostic values and immunological functions of PPA2, with a spotlight on breast cancer
Source: Front Immunol. 2024 Aug 8;15:1435502. doi: 10.3389/fimmu.2024.1435502 (PMC11338811; doi:10.3389/fimmu.2024.1435502)

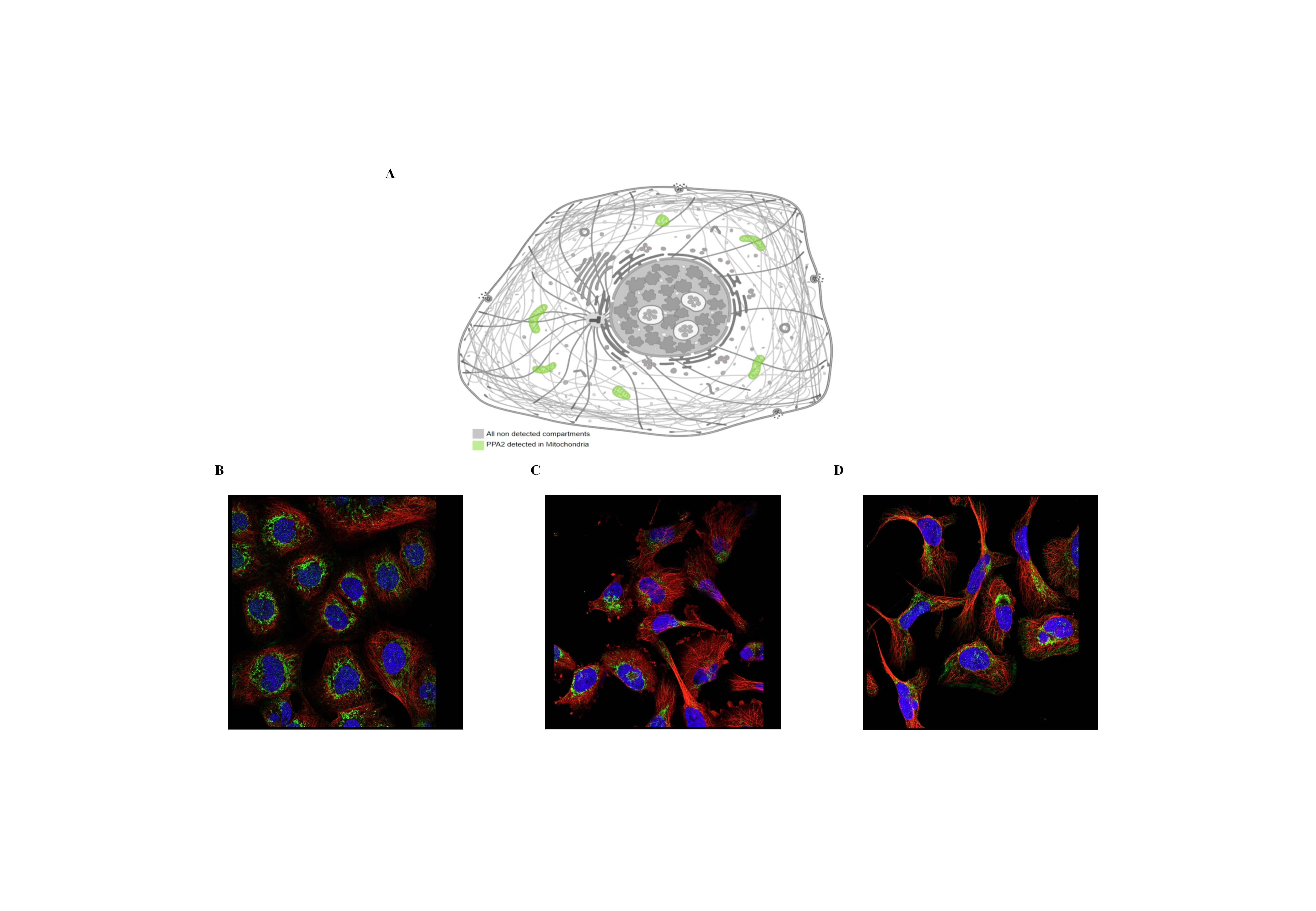

Supplement: Supplementary file 1 [file Image_1.jpeg]

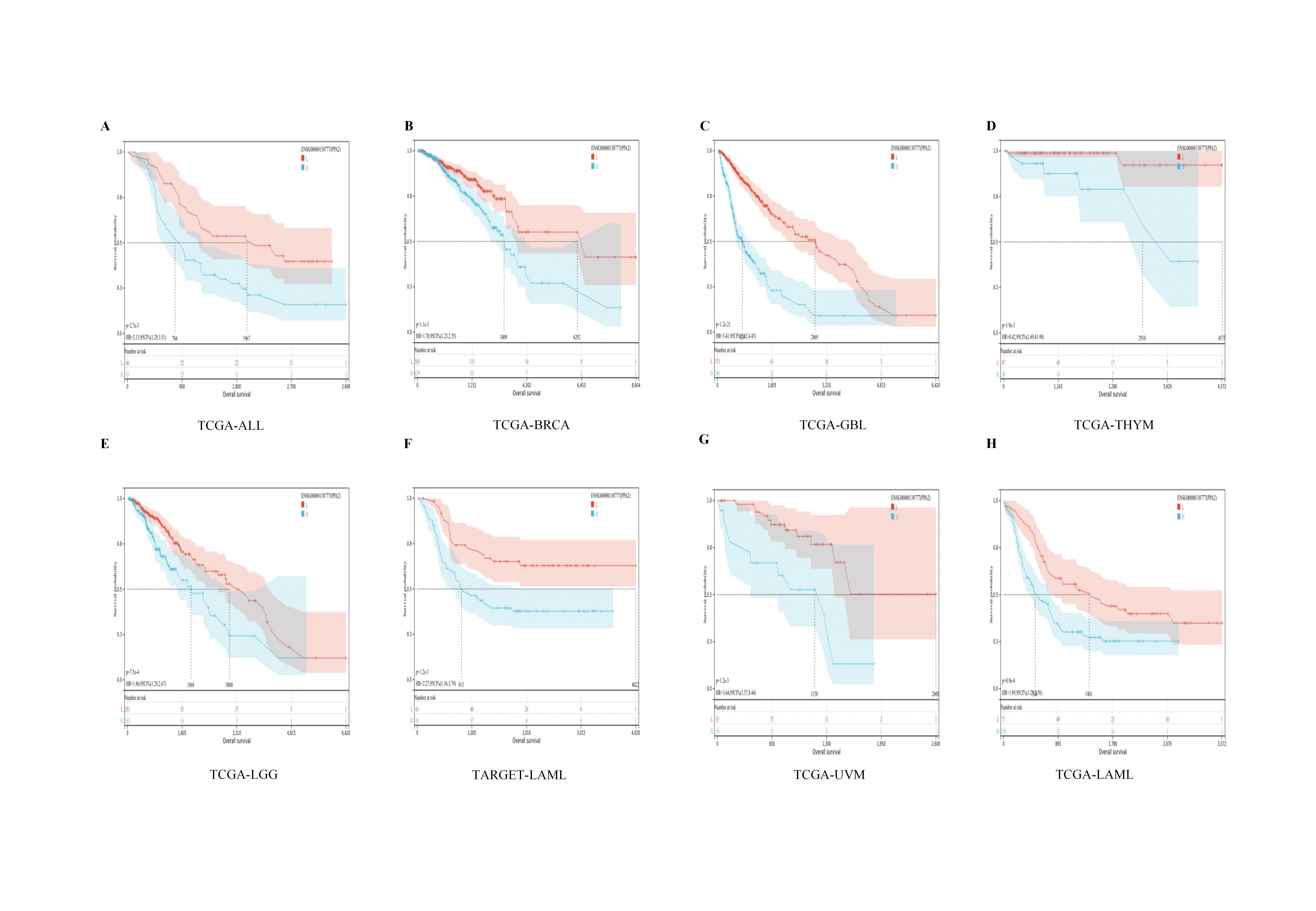

Supplement: Supplementary file 2 [file Image_2.jpeg]

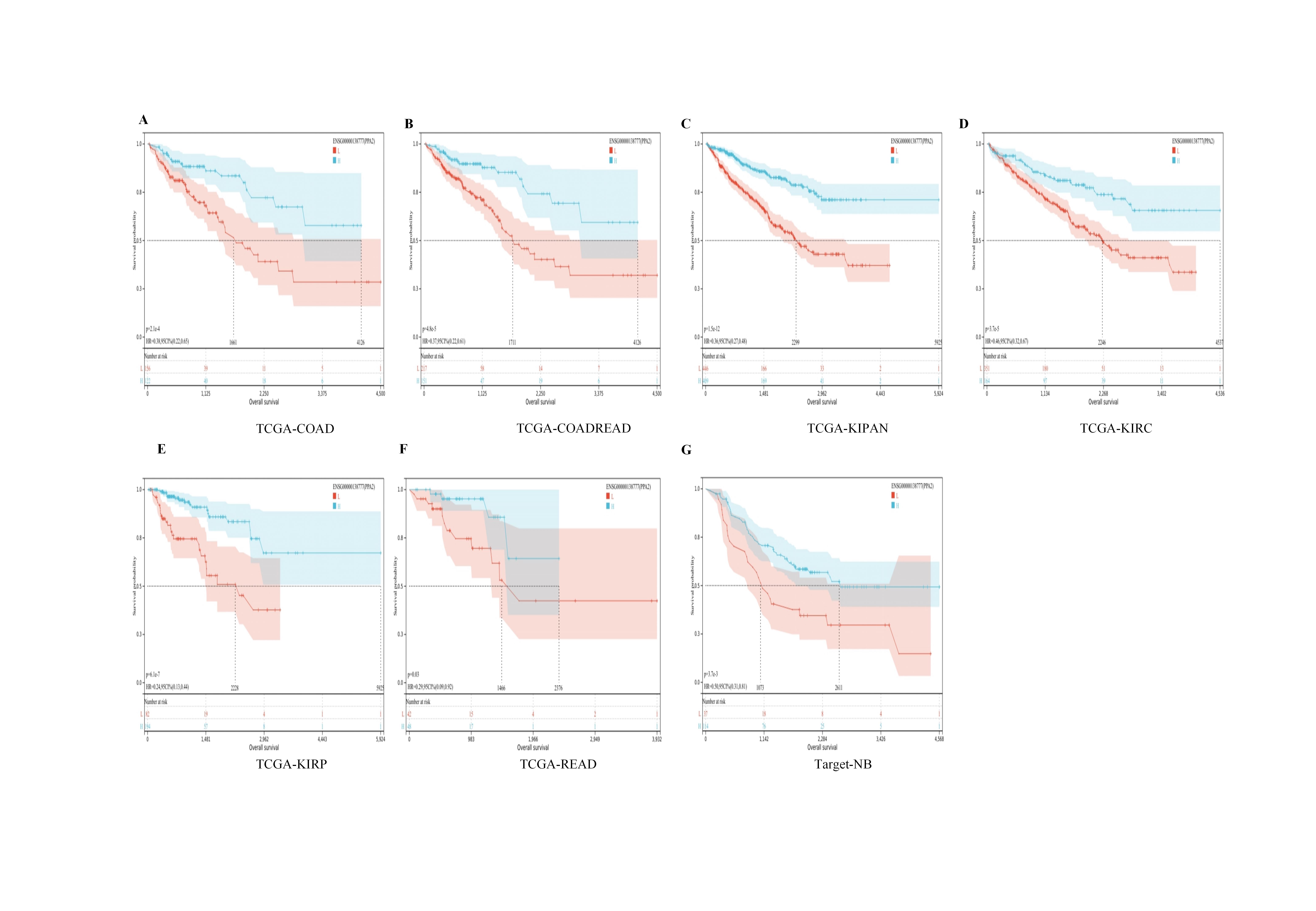

Supplement: Supplementary file 3 [file Image_3.jpeg]

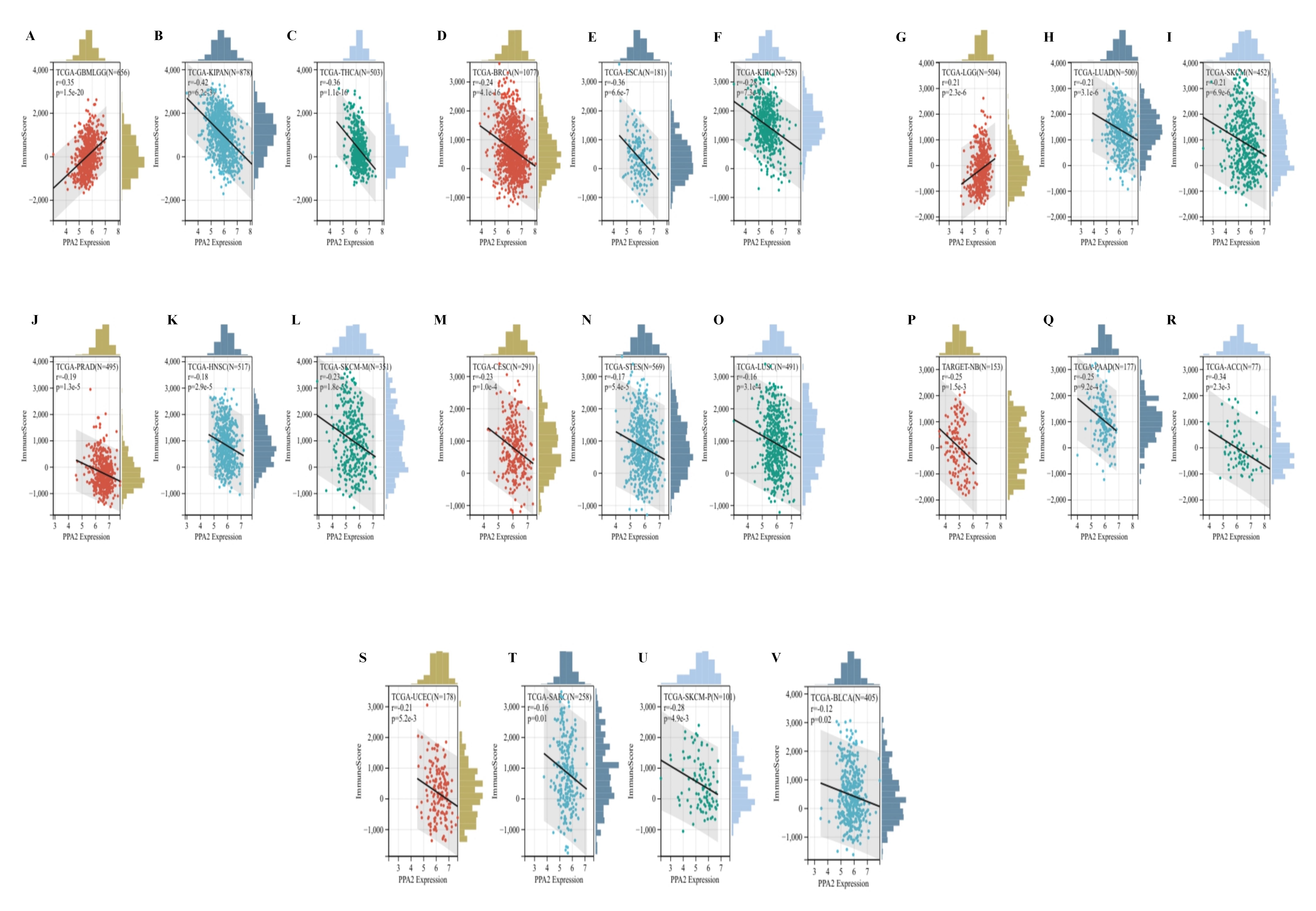

Supplement: Supplementary file 4 [file Image_4.jpeg]

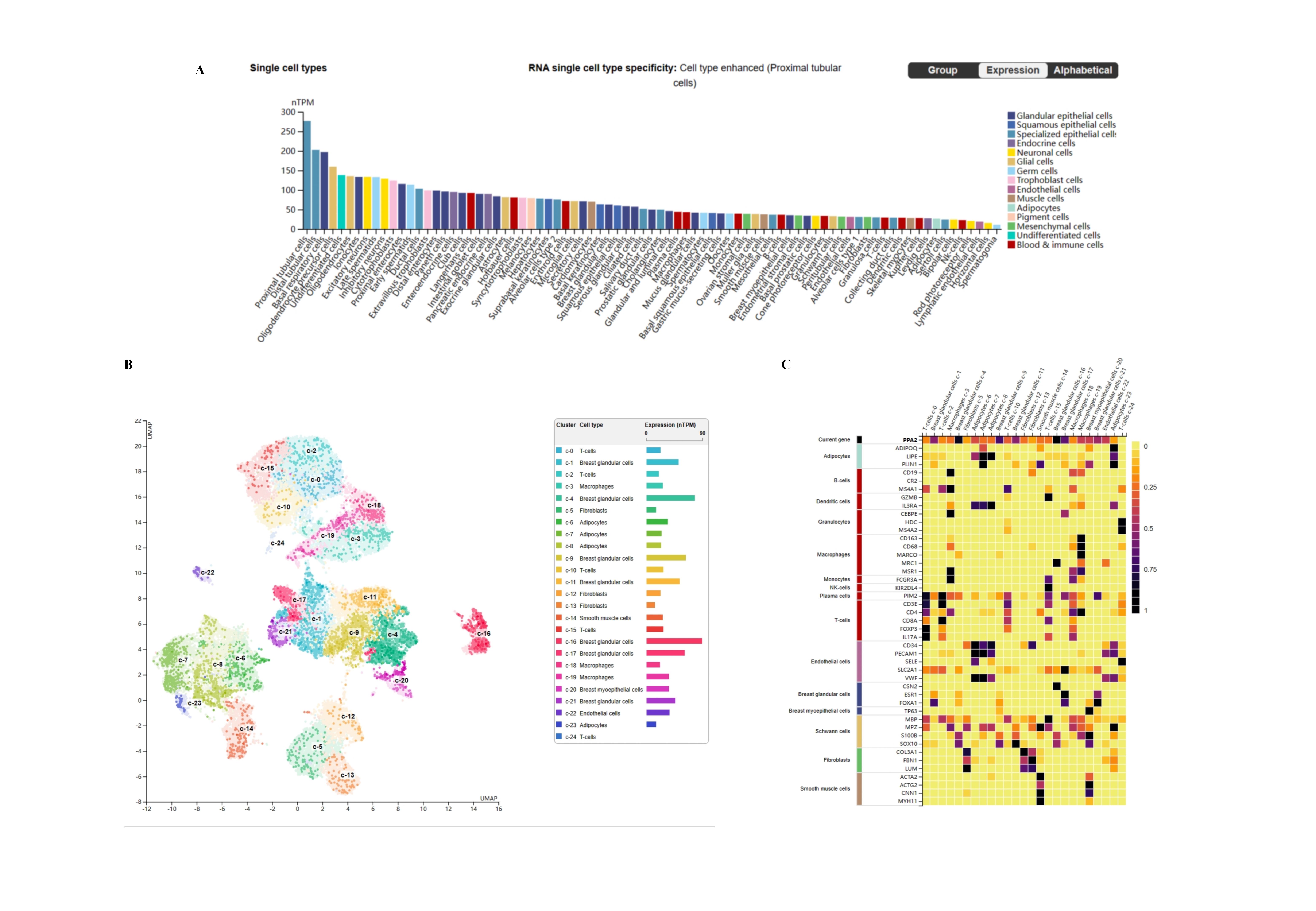

Supplement: Supplementary file 5 [file Image_5.jpeg]

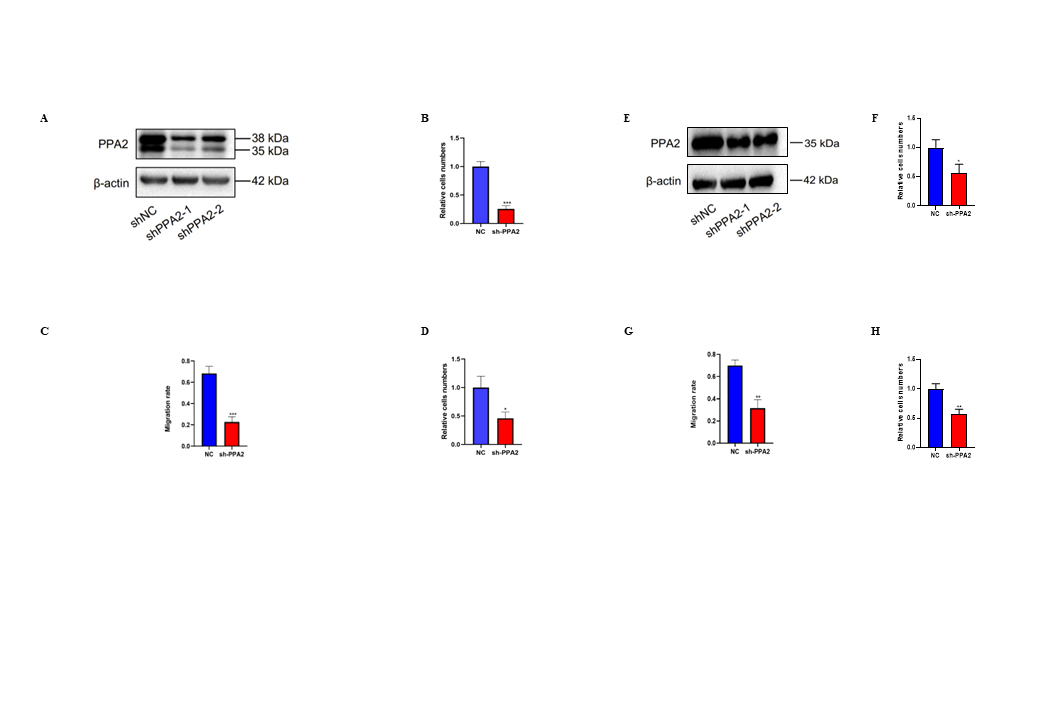

Supplement: Supplementary file 6 [file Image_6.tif]
